# Security Enhanced User Authentication Protocol for Wireless Sensor Networks Using Elliptic Curves Cryptography

**DOI:** 10.3390/s140610081

**Published:** 2014-06-10

**Authors:** Younsung Choi, Donghoon Lee, Jiye Kim, Jaewook Jung, Junghyun Nam, Dongho Won

**Affiliations:** 1 College of Information and Communication Engineering, Sungkyunkwan University, Jangangu, Suwonsi, Gyeonggido 440-746, Korea; E-Mails: yschoi@security.re.kr (Y.C.); dhlee@security.re.kr (D.L.); jykim@security.re.kr (J.K.); jwjung@security.re.kr (J.J.); 2 Department of Computer Engineering, Konkuk University, 268 Chungwondaero, Chungju, Chungcheongbukdo 380-701, Korea; E-Mail: jhnam@kku.ac.kr

**Keywords:** authentication protocol, elliptic curves cryptography, wireless sensor network

## Abstract

Wireless sensor networks (WSNs) consist of sensors, gateways and users. Sensors are widely distributed to monitor various conditions, such as temperature, sound, speed and pressure but they have limited computational ability and energy. To reduce the resource use of sensors and enhance the security of WSNs, various user authentication protocols have been proposed. In 2011, Yeh *et al.* first proposed a user authentication protocol based on elliptic curve cryptography (ECC) for WSNs. However, it turned out that Yeh *et al.*'s protocol does not provide mutual authentication, perfect forward secrecy, and key agreement between the user and sensor. Later in 2013, Shi *et al.* proposed a new user authentication protocol that improves both security and efficiency of Yeh *et al.*'s protocol. However, Shi *et al.*'s improvement introduces other security weaknesses. In this paper, we show that Shi *et al.*'s improved protocol is vulnerable to session key attack, stolen smart card attack, and sensor energy exhausting attack. In addition, we propose a new, security-enhanced user authentication protocol using ECC for WSNs.

## 1. Introduction

Wireless sensor networks (WSNs) provide a feasible real-time monitoring system. Wireless sensors can be easily deployed in various environments such as military surveillance, forest fire detection, health care and wildlife monitoring. WSNs basically consist of users, sensors and gateways whose communication security is a significant concern in real-world applications [1]. Users and gateways have sufficient resources to be used in the system, but sensors are different. Sensors have limited computational ability, low battery, low bandwidth, and a small amount of memory. Therefore, in WSNs, it is important to reduce the use of sensors to extend their lifespans [2–4].

Various user authentication protocols have been proposed for securing WSNs while minimizing the use of sensors. In 2004, Watro *et al.* proposed a user authentication protocol employing the RSA and Diffie-Hellman algorithms [5]. In 2006, Wong *et al.* proposed an efficient dynamic user authentication protocol using a hash function [6]. However, Tseng *et al.* demonstrated that Wong *et al.*'s authentication protocol is vulnerable to stolen-verifier attack, replay attack and forgery attack [7,8]. Later in 2009, Das proposed a two-factor user authentication protocol using smart cards. Das showed how to design an authentication protocol where only the user who is in possession of both the smart card and the password can pass the verification of the gateway [8]. However, several security-related flaws in Das's protocol have been disclosed by later studies as summarized below:

- He *et al.* demonstrated that Das's protocol is vulnerable to insider attacks and impersonation attacks, and that it does not allow users to change their passwords freely. He *et al.* proposed an improved two-factor protocol [9] which can resist insider and impersonation attacks.
- Khan and Alghathbar showed that Das's protocol fails to provide mutual authentication between the gateway and the sensor, and due to this failure, it is not secure against a gateway bypassing attack and a privileged-insider attack [10].
- Chen *et al.* also pointed out that Das's protocol does not achieve mutual authentication between the gateway and the sensor, and proposed a robust authentication protocol that provides the property of mutual authentication [11].

In 2011, Yeh *et al.* [2] revealed that Chen *et al.*'s protocol has difficulty in updating users' passwords and is vulnerable to an insider attack. As an improvement of Chen *et al.*'s protocol, Yeh *et al.* presented the first user authentication protocol that uses elliptic curve cryptography (ECC) in WSN environments. However, Han [12] showed that Yeh *et al.*'s protocol has still some security weaknesses: it does not provide perfect forward secrecy and fails to achieve mutual authentication and key agreement between the user and the sensor. To address these problems with Yeh *et al.*'s protocol, Shi *et al.* [3] have recently proposed a new smart-card-based user authentication protocol using ECC for WSNs. Shi *et al.*'s protocol performs more efficiently, both in terms of computation and communication costs, and provides better security than Yeh *et al.*'s protocol. However, we found that Shi *et al.*'s improvement is not secure enough yet and their protocol is susceptible to session key attacks, stolen smart card attacks, and sensor energy exhausting attacks. In addition to reporting the security weaknesses, we also show how to enhance the security of Shi *et al.*'s protocol with no significant increase in communication and computation costs. We analyze and verify the security of the proposed protocol using non-monotonic cryptographic logic (Rubin logic).

Throughout the paper, we make the following assumptions on the capabilities of the probabilistic polynomial-time adversary 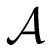 in order to properly capture security requirements of two-factor authentication protocols using smart cards in wireless sensor networks.

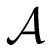
 has the complete control of all message exchanges between the protocol participants: a user, a sensor and the gateway. That is, 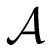 can eavesdrop, insert, modify, intercept, and delete messages exchanged among the three parties at will.

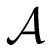
 is able to (1) extract the sensitive information on the smart card of a user through a power analysis attack [13,14] or (2) find out the user's password possibly via shoulder-surfing or by employing a malicious card reader. However, it is assumed that 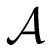 is unable to compromise both the two factors: the information on the smart card and the password of the user; it is clear that there is no way to prevent 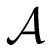 from impersonating the user if both factors are compromised.

## 2. Overview of Elliptic Curves Cryptography

In 1985, Neal Koblitz and Victor S. Miller proposed the use of elliptic curves in cryptography. After various studies on ECC, it has been widely used since the early 21st century. ECC is a type of public-key cryptography and based on the algebraic structure of elliptic curves over finite fields. Elliptic curves are also used in several integer factorization algorithms. ECC provides the important benefit of a smaller key size, despite which it is able to maintain the same degree of security as other types of public-key cryptography, such as RSA, DH and DSA. Therefore, ECC is especially useful for wireless devices, which typically have limited CPU capacity, power and network connectivity. Table 1 shows the NIST guidelines on choosing key sizes in ECC and other public key cryptography [15].

ECC has three related mathematical problems: the Elliptic Curve Discrete Logarithm Problem (ECDLP), Elliptic Curve Computational Diffie-Hellman Problem (ECCDHP), and Elliptic Curve Decisional Diffie-Hellman Problem (ECDDHP). No polynomial time algorithm can solve the ECDLP, ECCDHP and ECDDHP with non-negligible probability.

Let *p* > 3 be a large prime and choose two field elements *a, b* ∈ **F***_p_* satisfying 4*a*^3^ + 27*b*^2^ ≠ 0 mod *p* to define the equation of a non-supersingular elliptic curve **E**: *y*^2^ = *x*^3^ + *ax* + *b* mod *p* over **F***_p_*. Choose a generator point *P* = (*xP, yP*) whose order is a large prime number *q* over **E**(**F***_p_*). In the same way, a subgroup **G** of the elliptic curve group **E**(**F***_p_*) with order *q* is constructed. Then, the three mathematical problems in ECC are defined at various study [16–18] as follows.

- ECDLP: Given a point element *Q* in **G**, find an integer *x* ∈ ${\text{Z}}_{q}^{*}$ such that *Q* = *xP*, where *xP* indicates that the point *P* is added to itself *x* times by the elliptic curves operation.
- ECCDHP: For *a, b* ∈ ${\text{Z}}_{q}^{*}$, given two point elements *aP, bP* in **G**, compute *abP* in **G**.
- ECDDHP: For *a, b, c* ∈ ${\text{Z}}_{q}^{*}$, given three point elements *aP, bP* and *cP* in **G**, decide whether *cP* = *abP*.

## 3. Review of Shi *et al.*'s Protocol

In Shi *et al.*'s protocol [3], the gateway is a trusted node that holds two sufficiently large master keys, *x* and *y*. Before starting the system, the gateway and the sensors share a long-term secret key *SK_GS_* = *h*(*ID_S_n__*‖*y*). Shi *et al.*'s protocol consists of four phases; user registration phase, login phase, authentication phase, and password update phase. For convenience, the notations used throughout this paper are summarized in Table 2.

### 3.1. Registration Phase

**Figure 1.**
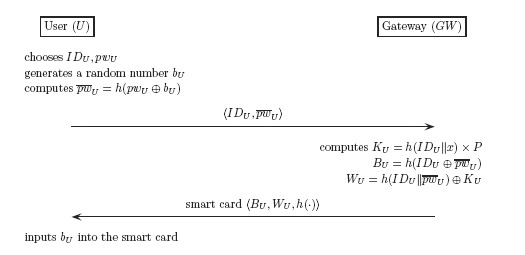
The registration phase of Shi *et al.*'s protocol.

In this phase, the user *U* securely submits its identity *ID_U_* and password *pw_U_* to the gateway *GW*. Then, *GW* issues *U* a smart card containing the user authentication information, as shown in Figure 1.

### 3.2. Login and Authentication Phases

**Figure 2.**
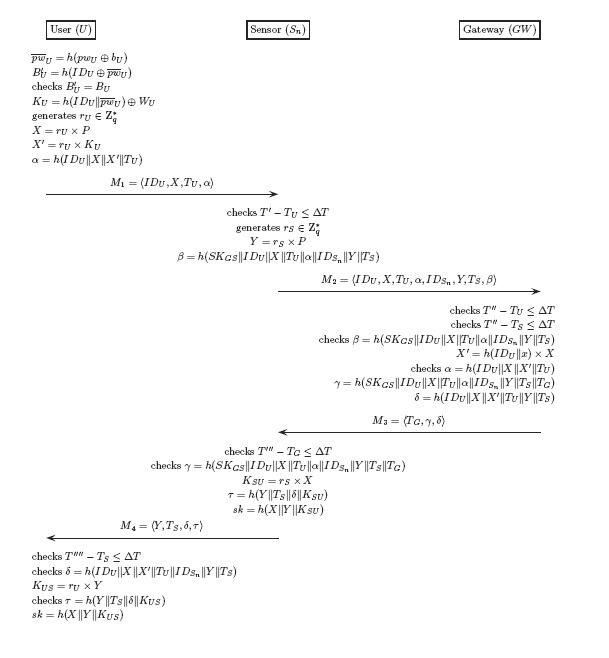
The login and authentication phases of Shi *et al.*'s protocol.

In the login and authentication phases, when *U* enters *ID_U_* and *pw_U_* into a smart card terminal, the smart card must validate the legitimacy of *U*. Then, *U, S_n_* and *GW* authenticate with each other. This protocol uses 4 messages (*M*_1_, *M*_2_, *M*_3_, *M*_4_) for mutual authentication, as described in Figure 2. Lastly, U and *S_n_* share the session key *sk*. After the authentication phase, *U* and *S_n_* communicate with each other using the session key *sk*.

### 3.3. Password Update Phase

**Figure 3.**
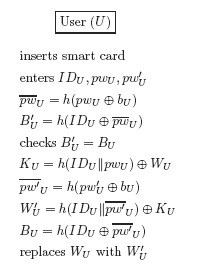
The password update phase of Shi *et al.*'s protocol.

In the password update phase, *U* enters the identity *ID_U_*, the old password *pw_U_*, and the new password $pw_{U}^{\prime }$. Then, the smart card updates the password after first checking the correctness of the old password, as shown in Figure 3.

## 4. Security Weaknesses in Shi *et al.*'s Protocol

This section shows that Shi *et al.*'s protocol is vulnerable to a session key attack, a stolen smart card attack, and a sensor energy exhausting attack.

### 4.1. Session Key Attack

**Figure 4.**
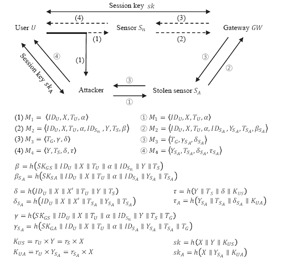
A session key attack on Shi *et al.*'s protocol.

In Shi *et al.*'s protocol, the user *U* and the sensor *S_n_* have to perform the login and authentication phases when they want to share a session key which will be used for protecting their subsequent communication. A problem occurs if *U* shares its session key with an attacker, not with the intended sensor *S_n_*. In the protocol, the gateway *GW* and the user *U* check each other's legitimacy using the authenticators *α* and *δ*, respectively. However, *α* and *δ* do not include information about the sensor *S_n_* with which *U* intends to establish a session key. The attacker exploits this design flaw in mounting a session key attack. The attack is depicted in Figure 4 and its description follows.

When *U* inputs *ID_U_* and *pw_U_*, and sends *M*_1_ to sensor *S_n_*, the attacker intercepts *M*_1_ and sends it to sensor *S_a_* which was previously stolen by the attacker. Upon receiving *M*_1_, the stolen sensor *S_a_* will generate the message *A*_2_ and send it to the gateway *GW*. However, the attacker replaces *ID_S_n__* contained in *A*_2_ with *ID_a_* to make *GW* believe that *ID_U_* wants to communicate with sensor *S_a_*, not with *S_n_*. After receiving *A*_2_, the gateway *GW* generates *A*_3_ without noticing any discrepancy and sends it to sensor *S_a_*. Lastly, the attacker sends the user *U* the message *A*_4_ generated by *S_a_* using the message *A_3_* from *GW*. Because there is no information about the sensor *S_n_* in *A*_4_ and *M*_4_, the user *U* undoubtedly shares the session key with the attacker while thinking that it has shared the key with the sensor *S_n_*.

### 4.2. Stolen Smart Card Attack

Kocher *et al.* and Messerges *et al.* pointed out that the confidential information stored in smart cards could be extracted by physically monitoring its power consumption [13,14]. Therefore, it is fair to say that if a user loses his or her smart card, all information in the smart card may be revealed to the attacker.

**Figure 5.**
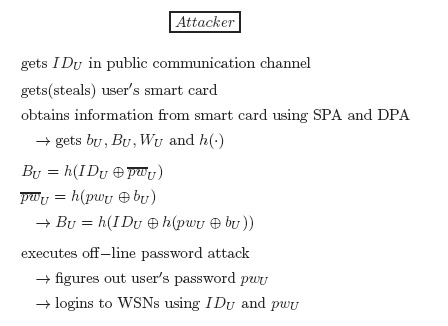
A stolen smart card attack on Shi *et al.*'s protocol.

In Shi *et al.*'s protocol, the smart card stores various information for user login and authentication. The smart card for the user *ID_U_* includes *b_U_, B_U_, W*_U_ and *h*(·). Using these information and *ID_U_*, an attacker can guess *U*'s password *pw_U_*. If *ID_U_* is used in public communication, the attacker can obtain or steal it without difficulty. Figure 5 describes a stolen smart card attack against Shi *et al.*'s protocol.

The attacker can obtain information from the smart card using attacks such as simple power analysis (SPA) and differential power analysis (DPA). This information includes *b_U_, B_U_, W_U_* and *h*(·). Recall that *B_U_* = *h*(*ID_U_* ⊕ *h*(*pw_U_* ⊕ *b_U_*)). Using *B_U_* as a password verifier, the attacker can easily find out the password *pw_U_* by mounting an offline password guessing attack (also known as an *offline dictionary attack*) [19–22] if the password *pw_U_* is not long enough. After successfully mounting the password guessing attack, the attacker can login and authenticate with the sensor *S_n_* and the gateway *GW* using the identity *ID_U_* and the password *pw_U_*.

### 4.3. Sensor Energy Exhausting Attack

The computational cost of a sensor is a critical consideration in the design of WSNs as it increases the consumption of the battery power of the sensor. Often it is economically advantageous to discard a sensor rather than recharge it. For this reason, the battery power of a sensor is usually important in wireless devices, with its lifetime determining the sensor lifetime. Previous work have suggested several types of energy exhausting attacks. Buttyan *et al.* [23] investigated the reliability of transport protocols for WSNs. Brownfield *et al.* [24] researched the battery depletion effect through the reduction of sleep cycles. Khouzani *et al.* [25,26] investigated malware attacks in battery-constrained wireless networks. As shown by the previous researches, WSNs need to eliminate unnecessary computational costs of sensors so that the effects of an energy exhausting attack on sensors can be minimized.

In Shi *et al.*'s protocol, the sensor performs various cryptographic operations such as one-way hash function evaluations, scalar-point multiplications, random number generations, and map-to-point hash function evaluations. Scalar-point multiplications are much more expensive than hash function evaluations. The computational costs of generating a random number and evaluating a map-to-point hash function are about half the cost of performing a scalar-point multiplication. A sensor consumes a large amount of energy to perform a scalar-point multiplication and very little to perform a hash function evaluation [27–29].

**Figure 6.**
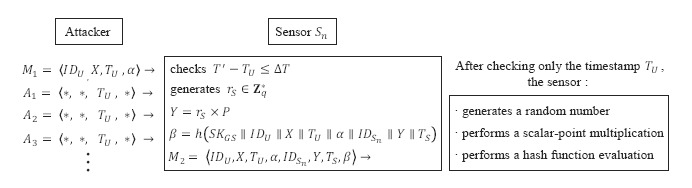
A sensor energy exhausting attack on Shi *et al.*'s protocol.

Figure 6 shows the possibility of a sensor energy exhaustion attack. The attacker can keep sending malicious messages, *A*_1_, *A*_2_, *A*_3_, generated to consume the battery power of the sensor. The attacker can do so because the sensor only checks the freshness of the timestamp in *M*_1_. For each of these fake messages, the sensor checks the freshness of the timestamp and proceeds to perform the subsequent cryptographic operations, thereby consuming large amounts of energy. Accordingly, it is necessary to modify the protocol so that the sensor can check if the message *M*_1_ is from a legitimate user, not from an imposter.

## 5. The Proposed Protocol

Like Shi *et al.*'s protocol, our proposed protocol is divided into three phases: the user registration phase, login and authentication phase, and password update phase. Before the protocol is ever executed, the gateway generates two master keys, *x* and *y*, and shares a long-term secret key *SK_GS_* = *h*(*ID_S_n__* ‖ *y*) with the sensor *S_n_*. In describing the protocol, we use the same notations as in Table 2 unless stated otherwise.

### 5.1. Registration Phase

**Figure 7.**
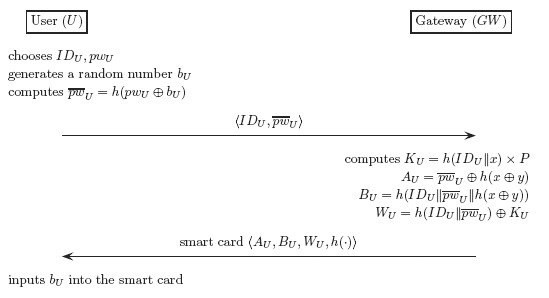
The registration phase.

For a user *U*, this phase is performed only once when *U* registers itself with the gateway *GW*. Figure 7 illustrates how the phase works, and its description follows:

(1) The user *U* chooses its identity *ID_U_* and password *pw_U_* freely, generates a random number *b_U_*, and computes ${\bar{pw}}_{U}=h\left(pw_{U}⊕b_{U}\right)$. *U* sends *ID_U_* and ${\bar{pw}}_{U}$ to *GW* via a secure channel.
(2) The gateway *GW* computes:
  $$\begin{array}{lll} K_{U} & = & h\left(ID_{U}\|x\right)\times P\\ A_{U} & = & {\bar{pw}}_{U}⊕h\left(x⊕y\right)\\ B_{U} & = & h\left(ID_{U}\left\|{\bar{pw}}_{U}\right\|h\left(x⊕y\right)\right)\\ W_{U} & = & h\left(ID_{U}\left\|{\bar{pw}}_{U}\right\|\right)⊕K_{U} \end{array}$$
  Then, *GW* issues *U* a smart card loaded with {*A_U_, B_U_, W_U_, h*(·)}.
(3) Lastly, *U* inputs the random number *b_U_* into the smart card.

### 5.2. Login and Authentication Phase

**Figure 8.**
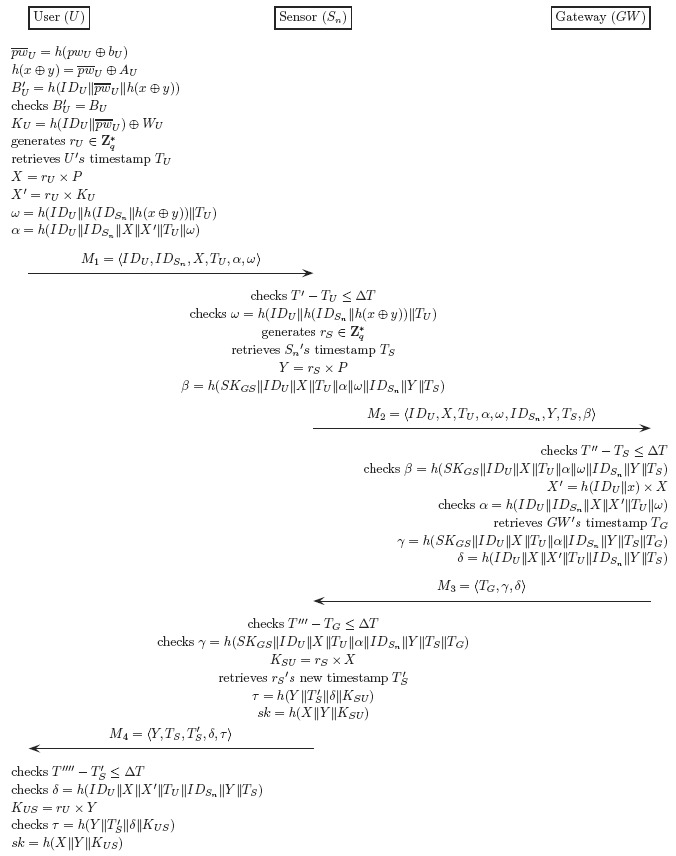
The login and authentication phase.

This phase is carried out whenever *U* wants to gain access to the WSN. During the phase, *U* establishes a session key with the sensor *S_n_* while being authenticated by the gateway *GW*. The phase proceeds as follows (see also Figure 8):

Step 1. *U* inserts its smart card into the card reader and inputs its identity *ID_U_* and password *pw_U_*. Then, the smart card computes:
  $$\begin{array}{cc} {\bar{pw}}_{U} & =h\left(pw_{U}⊕b_{U}\right)\\ B_{U}^{\prime } & =h\left(ID_{U}\left\|{\bar{pw}}_{U}\right\|h\left(x⊕y\right)\right) \end{array}$$
  and checks if *B_U_* is equal to $B_{U}^{\prime }$. If not equal, the smart card aborts the protocol. Otherwise, it retrieves the current timestamp *T_U_*, chooses a random number *r_U_* ∈ ${\mathbb{Z}}_{q}^{*}$, and computes:
  $$\begin{array}{cc} K_{U} & =h\left(ID_{U}\|{\bar{pw}}_{U}\right)⊕W_{U}\\ X & =r_{U}\times P\\ X^{\prime } & =r_{U}\times K_{U}\\ \omega & =h\left(ID_{U}\|h(ID_{S_{n}}\left\|h\left(x⊕y\right)\right\|T_{U}\right)\\ \alpha & =h\left(ID_{U}\left\|ID_{S_{n}}\right\|X\|X\prime \left\|T_{U}\right\|\omega \right) \end{array}$$
  After the computations, the smart card sends the message *M*_1_ = 〈*ID_U_, ID_S_n__, X, T_U_, α, ω*〉 to the sensor *S_n_*.
Step 2. Upon receiving *M*_1_ from *U*, the sensor *S_n_* retrieves the current timestamp *T′* and verifies the freshness of *U*'s timestamp *T_U_* by checking that:
  $$T^{\prime }-T_{U}\leq \Delta T$$
  where Δ*T* is the maximum allowed time difference between *T_U_* and *T′*. If *T_U_* is not fresh, *S_n_* rejects *U*'s request and aborts the protocol. Otherwise, *S_n_* checks if *ω* is equal to the hash value *h*(*ID_U_*‖*h*(*IDs_n_*‖*h*(*x* ⊕ *y*)) ‖ *T_U_*). If they are not equal, *S_n_* aborts the protocol. Otherwise, *S_n_* generates a random number *r_S_* ∈ $Z_{q}^{*}$ retrieves the current timestamp *T_S_*, and computes:
  $$\begin{array}{ll} Y & =r_{S}\times P\\ \beta & =h\left(SK_{GS}\left\|ID_{U}\right\|X\left\|T_{U}\right\|\alpha \left\|\omega \right\|ID_{S_{n}}\left\|Y\right\|T_{S}\right) \end{array}$$
  Then, *S_n_* sends the message *M*_2_ = 〈*ID_U_, X, T_U_, α, ω, ID_S_n__, Y, T_S_, β*〉 to the gateway *GW*.
Step 3. After receiving *M*_2_, *GW* retrieves the current timestamp *T″* and verifies the freshness of the timestamp *T_S_* by checking that *T″* − *T_S_* ≤ Δ*T*. If *T_S_* is not fresh, *GW* aborts the protocol. Otherwise, *GW* computes *X′* = *h*(*ID_U_*‖*x*) × *X* and checks if *α* equals *h*(*ID_U_*‖*ID_S_n__*‖*X*‖*X′*‖*T_U_*‖*ω*) and *β* equals *h*(*SK_GS_*‖*ID_U_*‖*X*‖*T_U_*‖*α*‖*ω*‖*ID_S_n__*‖*Y*‖*T_S_*). If either of the checks fails, *GW* aborts the protocol. Otherwise, *GW* retrieves the current timestamp *T_G_* and computes:
  $$\begin{array}{ll} \gamma & =h\left(SK_{GS}\left\|ID_{U}\right\|X\left\|T_{U}\right\|\alpha \left\|ID_{S_{n}}\right\|Y\left\|T_{S}\right\|T_{G}\right)\\ \delta & =h\left(ID_{U}\left\|X\right\|X^{\prime }\left\|T_{U}\right\|ID_{S_{n}}\left\|Y\right\|T_{S}\right) \end{array}$$
  Then, *GW* sends *M*_3_ = 〈*T_G_, γ, δ*〉 to the sensor *S_n_*.
Step 4. Having received *M*_3_, *S_n_* retrieves the current timestamp *T‴* and checks if *T‴* − *T_G_* ≤ Δ*T* and *γ* = *h*(*SK_GS_*‖*ID_U_*‖*X*‖*T_U_*‖*α*‖*ID_S_n__*‖*Y*‖*T_S_*‖*T_G_*) Only if both the checks hold, *S_n_* retrieves the new timestamp $T_{S}^{\prime }$ and computes:
  $$\begin{array}{cc} K_{SU} & =r_{S}\times X\\ \tau & =h\left(Y\left\|T_{S}^{\prime }\right\|\delta \|K_{SU}\right)\\ sk & =h\left(X\left\|Y\right\|K_{SU}\right) \end{array}$$
  Then, *S_n_* sends *M*_4_ = 〈*Y, T_S_*, $T_{S}^{\prime }$, δ, *τ*〉 to the user *U*.
Step 5. With *M*_4_ in hand, *U* retrieves the current timestamp *T⁗*, computes *K_US_* = *r_U_* × *Y*, and checks if (1) *T⁗*− *T′_S_* ≤ Δ*T*; (2) *δ* = h(*ID_U_*‖*X*‖*X′* ‖*T_U_*‖*ID_S_n__*‖*Y*‖*T_S_*); and (3) $\tau =h\left(Y\left\|T_{S}^{\prime }\right\|\delta \|K_{US}\right)$. If any of the checks fail, *U* aborts the protocol. Otherwise, *U* computes:
  $$sk=h\left(X\left\|Y\right\|K_{US}\right)$$

### 5.3. Password Update Phase

**Figure 9.**
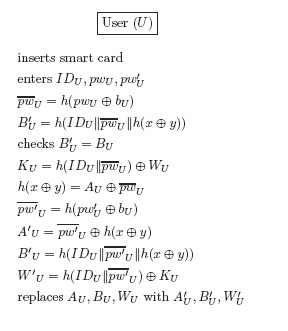
The password update phase.

Our protocol allows users to freely update their passwords. The password update phase works as follows (see also Figure 9):

1. The user *U* inserts its smart card into a smart card reader and enters the identity *ID_U_*, the old password *pw_U_*, and the new password *pw′_U_*.
2. The smart card computes ${\bar{pw}}_{U}=h\left(pw_{U}⊕b_{U}\right)$, $h\left(x⊕y\right)=A_{U}⊕{\bar{pw}}_{U}$, and $B_{U}^{\prime }=h\left(ID_{U}\left\|{\bar{pw}}_{U}\right\|h\left(x⊕y\right)\right)$ and checks if *B′* is equal to *B*. If they are not the same, the password update phase stops. Otherwise, the smart card computes:
  $$\begin{array}{cc} K_{U} & =W_{U}⊕h\left(ID_{U}\|{\bar{pw}}_{U}\right)\\ {\bar{pw^{\prime }}}_{U} & =h\left(p{w^{\prime }}_{U}⊕b_{U}\right)\\ {A^{\prime }}_{U} & ={\bar{pw^{\prime }}}_{U}⊕h\left(x⊕y\right)\\ {B^{\prime }}_{U} & =h\left(ID_{U}\left\|{\bar{pw^{\prime }}}_{U}\right\|h\left(x⊕y\right)\right)\\ {W^{\prime }}_{U} & =h(ID_{U}\left\|{\bar{pw^{\prime }}}_{U}\right)⊕K_{U} \end{array}$$
  and replaces *A_U_, B_U_* and *W_U_* with *A′_U_, B′_U_* and *W′_U_*, respectively.

## 6. Performance Comparison

Table 3 compares our improved protocol with Yeh *et al.*'s protocol [2] and Shi *et al.*'s protocol [3] in terms of the computational costs required by the protocols. The efficiency comparison is based on theoretical analysis and experimental results [3,27–29].

Notations used in Table 3 are described as follows:
*M*scalar-point multiplication*R*random point generation*A*point addition*P*map-to-point hash function evaluation*H*hash function evaluation

The computational costs of generating a random point and evaluating a map-to-point hash function are about half the cost of performing a scalar-point multiplication. Hash function evaluations and point addition operations are often ignored in cost estimates since they are much faster than scalar-point multiplications. If we ignore hash function evaluations, the computational costs described in Table 3 can be estimated as in Table 4.

As shown in Tables 3 and 4, our proposed protocol and Shi *et al.*'s protocol are more efficient than Yeh *et al.*'s protocol, in terms of the computational costs of the sensor and the gateway. In WSNs, it is important to minimize the energy consumption of the sensor node. In this sense, it is fair to say that our protocol and Shi *et al.*'s protocol are better suited for WSNs than Yeh *et al.*'s protocol. The performance of our proposed protocol is similar to that of Shi *et al.*'s protocol. But, as we demonstrated in Section 4, Shi *et al.*'s protocol is vulnerable to a session key attack, a stolen smart card attack, and a sensor energy exhausting attack. Consequently, we can say that our protocol enhances the security of Shi *et al.*'s protocol while maintaining the efficiency of the protocol.

## 7. Security Analysis and Verification

In this section, we first provide a heuristic security analysis for the proposed protocol and then formally verify the security analysis by using Rubin logic.

### 7.1. Heuristic Security Analysis

#### 7.1.1. Stolen-Verifier Attack

In WSNs, an attacker may attempt to mount a stolen-verifier attack if the gateway stores a password verifier [30] and then, impersonate a legal user using the verifier stolen from the gateway. However, in our protocol, the gateway does not store a password verifier of any kind but stores only the master secret keys *x* and *y* which are used in computing:

$$\begin{array}{ll} SK_{GS} & =h\left(ID_{S_{n}}\|y\right)\\ X^{\prime } & =h\left(ID_{U}\|x\right)\times X \end{array}$$

#### 7.1.2. Insider Attack

An insider attack occurs when the gateway manager or system administrator can access a user's secret (e.g., user password) and then impersonate the user. However, in our protocol, the user *U* does not send a plain password to the gateway, but sends only the password-derived hash value ${\bar{pw}}_{U}=h\left(pw_{U}⊕b_{U}\right)$. Since *b_U_* is a sufficiently high-entropy random number, the gateway cannot learn the password *pw_U_* from the hash value ${\bar{pw}}_{U}$. In addition, the gateway does not manage any table for storing user passwords or their verifiers (e.g., an ID/password table) Therefore, an insider attack is not possible against our protocol.

#### 7.1.3. Replay Attack

In our protocol, each of the protocol messages (*M*_1_, *M*_2_, *M*_3_ and *M*_4_) accompanies at least one of the authenticators (*α, β, γ, δ, τ* and *ω*) which are generated using a timestamp (*T_U_, T_S_, T′_S_* or *T_G_*) as part of the hash input. The protocol participants (*U, S_n_* and *GW*) verify the authenticity of incoming messages by checking the freshness of the timestamps and the legitimacy of the authenticators. But, an attacker cannot compute any of the authenticators for a fresh timestamp without knowing an appropriate secret. Therefore, our proposed protocol is secure against replay attacks.

#### 7.1.4. Man-in-the-Middle Attack

It is impossible for an attacker to mount a man-in-the-middle attack against our proposed protocol. In a typical man-in-the-middle attack, an attacker intercepts the messages being exchanged between the communicating parties and instead, sends arbitrary messages for its own benefit impersonating one of them to the other. But, our protocol allow the parties to authenticate all the protocol messages with the authenticators *α, β, γ, δ, τ* and *ω*, and therefore, is secure against man-in-the-middle attacks.

#### 7.1.5. Gateway Impersonation Attack

An attacker cannot impersonate the gateway because it cannot forge the message:

$$M_{3}=\langle T_{G},\gamma ,\delta \rangle$$

To generate *γ* or *δ*, one needs to know either *SK_GS_* or *h*(*ID_U_*‖*x*). However, *h*(*ID_U_*‖*x*) is the secret shared only between the user and the gateway while *SK_GS_* is the secret shared between the sensor and the gateway. Therefore, it is impossible for an attacker to mount a gateway impersonation attack.

#### 7.1.6. User Impersonation Attack

It is impossible for an attacker to impersonate the user as it cannot forge the message:

$$M_{1}=\langle ID_{U},ID_{S_{n}},X,T_{U},\alpha ,\omega \rangle$$

The attacker should know *X′* to compute *α* and should know *h*(*x* ⊕ *y*) to compute *ω*. But, the attacker knows neither *X′* nor *h*(*x* ⊕ *y*) and therefore, cannot mount a user impersonation attack.

#### 7.1.7. Sensor Impersonation Attack

An attacker cannot impersonate the sensor because it can forge the messages *M*_2_ = 〈*ID_U_, X, T_U_, α, ω, ID_S_n__, Y, T_S_, β*〉 and *M*_4_ = 〈*Y, T_S_,T′_S_, δ, τ*〉. The attacker cannot compute *β* without knowing *SK_GS_* and cannot compute *δ* without knowing the secret *h*(*ID_U_*‖*x*). But, the attacker knows neither *SK_GS_* nor *x* and therefore, cannot mount a sensor impersonation attack.

#### 7.1.8. Mutual Authentication

Mutual authentication is an important security property that an authentication protocol should achieve [31,32]. Our proposed protocol provides mutual authentication among the three parties: the user, the sensor and the gateway.

- The gateway authenticates the user using *α* in *M*_2_.
- The gateway authenticates the sensor using *β* in *M*_2_.
- The sensor authenticates the gateway using *γ* in *M*_3_.
- The user authenticates the gateway using *δ* in *M*_4_.
- The user and the sensor authenticate each other via *δ* from the gateway.

This means that our protocol achieves mutual authentication.

#### 7.1.9. Perfect Forward Secrecy

Perfect forward secrecy means that a session key derived from a set of long-term keys will not be compromised even if one of the long-term keys is compromised in the future. The proposed protocol uses the session key *sk* = *h*(*X*‖*Y*‖*r_S_* × *X*) for the sensor and *sk* = *h*(*X*‖*Y*‖*r_U_* × *Y*) for the user. Even though *h*(*ID_U_*‖*x*) and *x* are compromised, an attacker cannot know *r_U_* or *r_S_*. Under the assumption that the ECCDHP problem is hard, the attacker cannot compute *r_S_* from *r_S_* × *X* and *r_U_* from *r_U_* × *Y*. Therefore, our protocol provides perfect forward secrecy.

#### 7.1.10. Key Agreement

The proposed protocol provides key agreement between the user and the sensor. To the session-key computation, the user contributes its random number *r_U_* while the sensor contributes its random number *r_S_*. It is straightforward to verify that *K_SU_* and *K_US_* are equal:

$$\begin{array}{ll} K_{SU} & =r_{S}\times X=r_{S}\times r_{U}\times P\\ K_{US} & =r_{U}\times Y=r_{U}\times r_{S}\times P \end{array}$$

Since *K_SU_* = *K_US_*, it is clear that the user and the sensor compute session keys of the same value:

$$\begin{array}{cc} sk & =h\left(X\left\|Y\right\|K_{US}\right)\\ & =h\left(X\left\|Y\right\|K_{SU}\right) \end{array}$$

#### 7.1.11. Session Key Attack

In our protocol:

- *α* is combined with two identities *ID_U_* and *ID_S_n__*, which indicates that the user *U* wants to communicate with the sensor *S_n_*,
- *δ* is also combined with *ID_U_* and *ID_S_n__*, which indicates that the gateway has authenticated both the user *ID_U_* and the sensor *ID_S_n__*.

But, no attacker can compute *α* and *δ*, and therefore, can share a session key with the user.

#### 7.1.12. Stolen Smart Card Attack

In Shi *et al.*'s protocol, the attacker can obtain *b_U_* and *B_U_* from the smart card and thus can use *B_U_* = *h*(*ID_U_* ⊕ *h*(*pw_U_* ⊕ *b_U_*)) as the password verifier in its offline dictionary attack. However, in our protocol, *B_U_* is computed as $B_{U}=h\left(ID_{U}\left\|{\bar{pw}}_{U}\right\|h\left(x⊕y\right)\right)$. Even if the attacker obtains *b_U_* and *B_U_* from the smart card, it cannot use *B_U_* as a password verifier since it does not know the hash value *h*(*x* ⊕ *y*). Therefore, no attacker can mount an offline dictionary attack against our protocol.

#### 7.1.13. Sensor Energy Exhausting Attack

In Shi *et al.*'s protocol, the sensor has to generate a random number and execute a scalar-point multiplication whenever it receives the message *M*_1_ from the user. Random number generations and scalar-point multiplications are expensive and exhaust a large amount of the sensor's energy. This makes Shi *et al.*'s protocol vulnerable to a sensor energy exhausting attack. However, in our protocol, the sensor first checks the validity of *ω* = *h*(*ID_U_*‖*h*(*ID_S_n__*‖*h*(*x* ⊕ *y*))‖*T_U_*) before generating a random number and performing a scalar-point multiplication. Checking the validity of *ω* only requires one hash function evaluation. Therefore, our proposed protocol is secure against a sensor energy exhausting attack.

Table 5 summarizes and compares the security of our protocol, Yeh *et al.*'s protocol, and Shi *et al.*'s protocol.

### 7.2. Rubin Logic Verification

We analyze the proposed protocol using Rubin logic which can be applicable in analyzing an authentication protocol. Rubin logic integrates protocol analysis with specification and uses the notions of global sets, local sets, and actions. As the protocol run is progressed, the possession and belief sets (specified by local sets) are modified for each principal by inference rules (specified by global sets) and actions [33,34]. As the possession and belief sets are modified, secret set and observers sets (specified by global sets) are modified as well.

#### Global Sets

The first step of the specification of any protocol using Rubin logic is to instantiate the global sets with values. Global sets are public to each principal in a protocol specification.

- Principal Set: This set contains the principals who participate in a protocol.
- Rule Set: This set contains inference rules for deriving new statements from existing assertions.
- Secret Set: This set contains all of the secrets that exist at any given time in the system.
- Observers Sets: For each secret, its set contains all the principals who could possibly know the secret by listening to network traffic or generating it themselves.

#### Local Sets

Local sets are private to each principal in a protocol specification [35]. For each principal, *P_i_*, Rubin logic defines the following sets:

- Possession Set(*P_i_*): This set contains all the data relevant to security that this principal knows or possesses. We denote this set by POSS(*P_i_*) = (*poss*_1_, *poss*_2_, ⋯, *poss_n_*).
- Belief Set(*P_i_*): This set contains all the beliefs hold by a principal. For example, the keys it holds between itself and other principals, beliefs about jurisdiction, beliefs about freshness, and beliefs about the possessions of other principals. We denote this set by BEL(*P_i_*) = (*bel*_1_, *bel*_2_, ⋯, *bel*_n_).
- Behavior List(*P_i_*): This item is a list rather than a set because the elements are ordered. BL(*P_i_*) = Behavior List of *P_i_*.

#### Actions

Rubin logic defines actions for dealing with the knowledge in a protocol [36]. The action lists that precede and follow message operations in a principal's behavior list determine a sequence of events performed by the principal during a protocol run. We use the following actions:

- Generate-nonce(*N*)
- Send(*P_i_, X*)
- Receive(*P_i_, X*)
- Update(*X*)
- Forget(*X*)
- Concat(*X*_1_, *X*_2_, ⋯,*X_n_*)
- XOR(*X*_1_, *X*_2_, ⋯,*X_n_*)
- Check(*X*_1_, *X*_2_, ⋯, *X_n_*)
- Scalar-multiplication(*X*_1_, *X*_2_, ⋯, *X_n_*)
- Hash(*h*(·); *X*_1_, *X*_2_, ⋯, *X_n_*)
- Check-freshness(*T*)

Here, Concat(*X*_1_, *X*_2_, ⋯, *X_n_*) is the action that concatenates the submessages *X*_1_, *X*_2_, ⋯, *X_n_*.

#### 7.2.1. Protocol Specification

Notations used for the protocol specification is the same as those in Table 2. Phases 1, 2 and 3 represent the registration phase, the login and authentication phase, and the password updated phase. The global and local sets for the protocol are specified as follows:

##### Global Sets

The global sets are specified as follows:

- Principal set: A principal is one of *U, S_n_* and *GW*. *U* is the protocol initiator.
- Rule set:
  – *X* contains *Y*: *Y* appears as a submessage of X.
  – *S* ≔← *f*(*S*):* S* is replaced by the value f(*S*).
  – *X* from *E*: *X* is received from *E*.
  – LINK(*N*): LINK is used to link responses to challenges. When a principal generates a nonce, *N*, the formula LINK(*N*) is added to the belief set of the principal.
- Secret Set: {*pw_U_, b_U_, x, y, h*(*x* ⊕ *y*), *SK_GS_*}
- Observers Sets:
  – Observers(*pw_U_*): {*U*}
  – Observers(*b_U_*): {*U*}
  – Observers(*x*): {*GW*}
  – Observers (*y*): {*GW*}
  – Observers(*h*(*x* ⊕ *y*)): {*S_n_,GW*}
  – Observers(*SK_GS_*): {*S_n_,GW*}

##### Local Sets

The local sets are defined for each *U, S_n_* and *GW*. Tables 6^7^ and 8 show the specification of the local sets for *U, S_n_* and *GW*, respectively.

#### 7.2.2. Analysis and Verification

In phase 1, *U* initiates the protocol, and then the actions in BL(*U*) are performed. Firstly, (U1)–(U3) actions in BL(*U*) are performed, which represent that *U* sends *ID_U_* and ${\bar{pw}}_{U}$ to *GW* for registration. Next, (GW1)–(GW8) actions in BL(*GW*) are performed to generate *A_U_, B_U_, K_U_* and *W_U_*, and to send them to *U*. By (GW8), *GW* deletes *ID_U_*, ${\bar{pw}}_{U}$, *A_U_, B_U_, K_U_* and *W_U_* from POSS(*GW*) and BEL(*GW*). Lastly, the (U4) action in BL(*U*) is executed, then phase 1 is finished. Due to the (GW8) forget action, the local sets of *GW* are not changed. However, the local sets of principal *U* are changed as described below.

- POSS(*U*) = {*pw_U_, b_U_*, ${\bar{pw}}_{U}$, {*ID_U_*}, {*A_U_, B_U_, W_U_, h*(·)} from *GW*}
- BEL(*U*) = {#(*pw_U_*), #(b*_U_*), #( ${\bar{pw}}_{U}$)}

Accordingly, the global sets are modified as follows:

- Secret set: {*pw_U_, b_U_*, ${\bar{pw}}_{U}$, *x, y, SK_GS_, h*(*x* ⊕ *y*)}
- Observers sets:
  – Observers ( ${\bar{pw}}_{U}$): {*U*}

In the (U5)–(U8) actions in BL(*U*) of phase 2, the smart card authenticates *U*, who inputs *ID_U_* and *pw_U_*, by checking whether *B_U_* and $B_{U}^{\prime }$ are same or not. Next, the (U9)–(U15) actions are executed to generate the protocol values *X, X′, h*(*x* ⊕ *y*), *ω, α* and *r_U_*. After the (U16) update action, the local sets of *U* are changed as follows:

- POSS(*U*) = {*ID_S_n__, pw_U_, b_U_*, ${\bar{pw}}_{U}$, *X, X′, h*(*x* ⊕ *y*), *T_U_, α, ω, r_U_*, {*ID_U_*}}
- BEL(*U*) = {#(*pw_U_*), #(*b_U_*), #(*r_U_*), #( ${\bar{pw}}_{U}$), #(*X′*), #(*h*(*x* ⊕ *y*)), #(*T_U_*), LINK(*r_U_*)}

Then, the global sets are modified as follows:

- Secret set: {*pw_U_, b_U_*, ${\bar{pw}}_{U}$, *x, y, X′, SK_GS_, h*(*x* ⊕ *y*)}
- Observers sets:
  – Observers(*X′*): {*U*}
  – Observers(*h*(*x* ⊕ *y*)): {*U*}

After the (U5)–(U16) actions are finished, *S_n_* starts the actions in BL(*S_n_*) with the incoming message *M*_1_ from *U*. The (SN1)–(SN3) actions in BL(*S_n_*) are performed to verify the correctness of message *M*_1_. If the check succeeds, the (SN4)–(SN8) actions are performed to make the values *Y, β* and *r_S_*, and to send the message *M*_2_. The local sets of *S_n_* are changed as follows.

- POSS(*S_n_*) = {*Y, T_S_, r_S_, β, SK_GS_, h*(*x* ⊕ *y*), {*ID_S_n__*}, {*ID_U_, X, T_U_, α, ω*} from *U*}
- BEL(*S_n_*) = {#(*r_S_*), #(*SK_GS_*), #(*h*(*x* ⊕ *y*)), #(*T_S_*), LINK(*r_S_*)}

In this case, the global sets remain unchanged and thus, the secret set is the same as above:

- Secret set: {*pw_U_, b_U_*, ${\bar{pw}}_{U}$, *x, y, X′, SK_GS_, h*(*x* ⊕ *y*)}

After (SN1)–(SN8) actions of *BL*(*S_n_*) are finished, (GW9)–(GW17) actions *of* BL(*GW*) are executed. (GW9)–(GW13) actions check the timestamp of *S_n_*, and then verify the legitimacy of *U* and *S_n_*. If they are correct, (GW14)–(GW17) actions of BL(*S_n_*) are executed to make values(*γ, δ*) for authentication and send message. *γ* is used for authentication with *S_n_* and *δ* is used for authentication with *U*.

After the (SN1)–(SN8) actions are done, the (GW9)–(GW13) actions in BL(*GW*) are performed to check the legitimacy of *U* and *S_n_*. If the verification succeeds, the (GW14)–(GW17) actions are performed to generate *γ* and *δ* and to send the message *M*_3_ to *S_n_*. The local sets of *GW* are modified as shown below.

- POSS(*GW*) = {*T_G_, γ, δ,x, y, X′, SK_GS_, h*(*x* ⊕ *y*), {*ID_U_, X, T_U_, α, ω, ID_S_n__, Y, T_S_, β*} from *S_n_*}
- BEL(*GW*) = {(#(*x*), #(*y*), #(*X′*), #(*SK_GS_*), #(*h*(*x* ⊕ *y*)), #(*T_G_*)}

The global sets are updated as follows:

- Secret set: {*pw_U_, b_U_*, ${\bar{pw}}_{U}$, *x, y, X′, SK_GS_, h*(*x* ⊕ *y*)}
- Observers sets:
  – Observers(*X′*) = {*GW*}

After the (GW9)–(GW17) actions are finished, the (SN9)-(SN11) actions in BL(*S_n_*) are conducted to verify the legitimacy of *GW* and *U* via the authenticator *γ*. If the verification process is completed, the (SN12)–(SN16) actions are performed to generate *τ* and *sk* from *r_S_, K_SU_, X* and *Y*, and to send the message *M*_4_ to *U*. The local sets of *S_n_* is updated as follows:

- POSS(*S_n_*) = {*Y*, $T_{S}^{\prime }$, *K_SU_, r_S_, τ, sk, SK_GS_*, {*ID_S_n__*},{*T_G_, γ, δ*} from *GW*}
- BEL(*S_n_*) = {#(*K_SU_*), #(*sk*), #(*SK_GS_*), #( $T_{S}^{\prime }$), LINK (*r_S_*)}

Accordingly, the global sets are modified as follows:

- Secret set: {*pw_U_, b_U_*, ${\bar{pw}}_{U}$, *x, y, K_SU_, sk, X′, SK_GS_, h*(*x* ⊕ *y*)}
- Observers sets:
  – Observers (*K_SU_*) = {*S_n_*}
  – Observers(*sk*) = {*S_n_*}

The (U17)–(U19) actions in BL(*U*) are to check the legitimacy of *GW* and *S_n_* while the (U20)–(U22) actions are to generate the session key *sk* from *r_U_, K_US_, X* and *Y*. So, the conditions for the linkage rule are satisfied.

- POSS(*U*) = {*K_US_, sk*, {*ID_U_*}, {*Y*, $T_{S}^{\prime }$, *δ, τ*} from *S_n_*}
- BEL(*U*) = {#(*K_US_*), *#*(*X′*), *#*(*sk*), *#*(*h*(*x* ⊕ *y*)}
- Secret set: {*pw_U_, b_U_*, ${\bar{pw}}_{U}$, *x, y, K_SU_, K_US_, sk, X′, SK_GS_*, (*x* ⊕ *y*)}
- Observers sets:
  – Observers (*K_US_*) = {*U*}
  – Observers(*sk*) = {*U*}

In phase 3, *U* changes its password and updates *A_U_, B_U_* and *W_U_* stored in the smart card. In this phase, the local sets of *U* and the global sets remain unchanged.

The following shows the final version of the global sets.

- Secret set: {*pw_U_, b_U_*, ${\bar{pw}}_{U}$, *x, y, K_SU_, K_US_, sk, X′, SK_GS_, h*(*x* ⊕ *y*)}
- Observers sets:
  – Observers (*pw_U_*): {*U*}
  – Observers (*b_U_*): {*U*}
  – Observers( ${\bar{pw}}_{U}$): {*U*}
  – Observers(*x*): {*GW*}
  – Observers(*y*): {*GW*}
  – Observers (*K_SU_*): {*S_n_*}
  – Observers (*K_US_*): {*U*}
  – Observers(*sk*): {*U, S_n_*}
  – Observers(*X′*): {*U,GW*}
  – Observers(*SK_GS_*): {*S_n_, GW*}
  – Observers(*h*(*x* ⊕ *y*)): {*U, S_n_, GW*}

This result implies that:

- *pw_U_, b_U_* and ${\bar{pw}}_{U}$ are known only to the user *U*.
- *x* and *y* are known only to the gateway *GW*.
- The long-term key *SK_GS_* shared between *S_n_* and *GW* is not exposed.
- *X′* is only known to *U* and *GW*.
- *K_US_* and *K_SU_* are only available to *U* and *S_n_*.
- The session key *sk* is securely shared between *U* and *S_n_*.
- *h*(*x* ⊕ *y*) is only known to the authorized principals: *U, S_n_* and *GW*.
- *U, S_n_* and *GW* are mutually authenticated during the protocol execution.

This verifies the security claims we made in the previous subsection.

## 8. Conclusions

In this paper, we have identified that Shi *et al.*'s ECC-based authentication protocol designed for wireless sensor networks (WSNs) is vulnerable to: a session key attack, a stolen smart card attack, and a sensor energy exhausting attack. We have also proposed a new authentication protocol that addresses the identified security weaknesses. Our proposed protocol is as efficient as Shi *et al.*'s protocol and is better suited for WSNs than Yeh *et al.*'s protocol, the predecessor of Shi *et al.*'s protocol. As for the security of the proposed protocol, we have provided a heuristic analysis and formally verified the analysis using Rubin logic.

## Figures and Tables

**Table 1.** ECC key sizes compared with other PKC schemes.

**Security (bits)**	**ECC**	**RSA/DH/DSA**	**MIPS-Years to Attack**	**Protection Lifetime**
80	160	1,024	10^12^	until 2010
112	224	2,048	10^24^	until 2030
128	256	3,072	10^28^	beyond 2031
192	384	7,680	10^47^	beyond 2031
256	512	15,360	10^66^	beyond 2031

**Table 2.** Notations.

**Symbol**	**Description**
*p, q*	Two large prime numbers
*F_P_*	A finite field
*E*	An elliptic curve defined on finite field *F_P_* with large order
*G*	The group of elliptic curve points on *E*
*ID_U_*	The identity of user *U*
*ID_S_n__*	The identity of sensor *S_n_*
*pw_U_*	The user *U*'s password
*GW*	The gateway of WSN
*x, y*	The master keys of *GW*
*h*(·)	A secure one-way hash function
‖	A string concatenation operation
⊕	A bitwise XOR operation

**Table 3.** Efficiency comparison.

**Protocol**	**Computational Cost**		

	**User**	**Sensor**	**Gateway**
Yeh *et al.*'s protocol	2*M* +1*R*+1*A*+4*H*	2*M* +1*R* +1*A*+ 1*P* +1*H*	3*M* +1*R*+1*P* + 1*H*
Shi *et al.*'s protocol	3*M* +5*H*	2*M* +3*H*	1*M* +4*H*
Our protocol	3*M* +7*H*	2*M* +4*H*	1*M* +4*H*

**Table 4.** Estimated efficiency comparison.

**Protocol**	**Computational Cost**		

	**User**	**Sensor**	**Gateway**
Yeh *et al.*'s protocol	2.5*M*	3*M*	3*M*
Shi *et al.*'s protocol	3*M*	2*M*	1*M*
Our protocol	3*M*	2*M*	1*M*

**Table 5.** Security comparison.

**Attack and Security Property**	**Yeh *et al.*'s Protocol**	**Shi *et al.*'s Protocol**	**Our Protocol**
Stolen-verifier attack	Secure	Secure	Secure
Insider attack	Secure	Secure	Secure
Replay attack	Secure	Secure	Secure
Man-in-the-middle attack	Secure	Secure	Secure
Gateway impersonation attack	Secure	Secure	Secure
User impersonation attack	Secure	Secure	Secure
Sensor impersonation attack	Insecure	Secure	Secure
Mutual authentication	No	Yes	Yes
Perfect forward secrecy	No	Yes	Yes
Key agreement between user and sensor	No	Yes	Yes
Session key attack	Insecure	Insecure	Secure
Stolen smart card attack	Insecure	Insecure	Secure
Sensor energy exhausting attack	Insecure	Insecure	Secure

**Table 6.** Local sets specification for principal *U*.

**Principal *U***	
POSS(U) = {*pw_U_, b_U_*, {*ID_U_*}}	(U16) Update(*ID_U_, ID_S_n__, X, T_U_, α, ω*)
BEL(*U*) = {#(*pw_U_*), #(*b_U_*)}	(U17) Receive(*S_n_*, {*Y, T_S_, δ, τ*})
BL(U)	(U18) Check-freshness(*T′_S_*)
Phase 1	(U19) Check (*δ*, Hash(*h*(·); Contat(*ID_U_, X, X′,T_U_, ID_S_n__, Y, T_S_*)))
(U1) ${\bar{\text{pw}}}_{U}\leftarrow \text{Hash}(h(\cdot );\text{XOR}(pw_{U},b_{U}))$	
(U2) Send (*GW*,{*ID_U_*, ${\bar{pw}}_{U}$})	(U20) *K_US_* ← Scalar-multiplication(*r_U_, Y*)
(U3) Update(*ID_U_*, ${\bar{pw}}_{U}$)	(U21) Check(*τ*, Hash(*h*(·); Contat(*Y, T′_S_, δ, K_US_*)))
(U4) Receive(*GW*, {*A_U_, B_U_, W_U_, h*(·)})	(U22) *sk* ← Hash(*h*(·); Contat(*X, Y, K_US_*))
Phase 2	Phase 3
(U5) ${\bar{\text{pw}}}_{U}\leftarrow \text{Hash}(h(\cdot );\text{XOR}(pw_{U},b_{U}))$	(U23) ${\bar{\text{pw}}}_{U}\leftarrow \text{Hash}(h(\cdot );\text{XOR}(pw_{U},b_{U}))$
(U6) *h*(*x*⊕*y*) ← XOR( ${\bar{pw}}_{U}$, *A_U_*)	(U24) *B′_U_* ← Hash(*h*(·); Concat (*ID_U_*, ${\bar{pw}}_{U}$, *h*(*x*(⊕ *y*))
(U7) *B′_U_* ← Hash(*h*(·);Concat (*ID_U_*, ${\bar{pw}}_{U}$, *h*(*x*⊕*y*)))	(U25) Check(*B′_U_, B_U_*)
(U8) *Check*(*B′_U_, B_U_*)	(U26) *K_U_* ← XOR(Hash(*h*(·); Concat(*ID_U_*, ${\bar{pw}}_{U}$)), *W_U_*)
(U9) *K_U_* ← XOR(Hash(*h*(·); Concat(*ID_U_*, ${\bar{pw}}_{U}$)),*W_U_*)	(U27) *h*(*x*(⊕ *y*) ← XOR( ${\bar{pw}}_{U}$, *A_U_*)
(U10) Generate-nonce(*r_U_*)	(U28) ${\bar{pw\prime }}_{U}\leftarrow \text{Hash}\left(h\left(\cdot \right);\text{XOR}\left(pw\prime_{U},A_{U}\right)\right)$
(U11) *X* ← Scalar-multiplication(*r_U_, P*)	(U29) *A′_U_* ← XOR ( ${\bar{pw}}_{U}$, *h*(*x* ⊕ *y*))
(U12) *X′* ← Scalar-multiplication(*r_U_, K_U_*)	(U30) *B′_U_* ← Hash(*h*(·); Concat(*ID_U_*, ${\bar{pw^{\prime }}}_{U}$, *h*(*x*(⊕ *y*)))
(U13) *ω* ← Hash(*h*(·); Concat(*ID_U_*, Hash(*h*(·);*ID_S_n__, h*(*x* ⊕ *y*)), *T_U_*))	(U31) *W′_U_* ← XOR(Hash(*h*(·); Concat(*ID_U_*, ${\bar{pw^{\prime }}}_{U}$)), *K_U_*)
	(U32) *A_U_* ← *A′_U_*
(U14) *α* ← Hash(*h*(·); Concat(*ID_U_, ID_S_n__, X, X′, T_U_,ω*))	(U33) *B_U_* ← *B′_U_*
(U15) Send(*S_n_*, {*ID_U_, ID_S_n__, X, T_U_, α, ω*})	(U34) *W_U_* ← *W′_U_*

**Table 7.** Local sets specification for principal *S_n_*.

**Principal *S_n_***	
POSS(*S_n_*) = {*SK_GS_, h*(*x* ⊕ *y*), {*ID_S_n__*}}	(SN7)
BEL(*S_n_*) = {#(*SK_GS_*), #(*h*(*x* ⊕ *y*))}	Send(*GW*, {*ID_U_, X, T_U_, α, ω, ID_S_n__, Y, T_S_, β*})
BL(*S_n_*)	(SN8) Update(*ID_U_, X, T_U_, α, ω, ID_S_n__, Y, T_S_, β*)
Phase 2	(SN9) Receive(*GW*, {*T_G_, γ, δ*})
(SN1) Receive(*U*, {*ID_U_, ID_S_n__, X, T_U_, α, ω*})	(SN10) Check-freshness(*T_G_*)
(SN2) Check-freshness(*T_U_*)	(SN11) Check
(SN3) Check	(*γ*,Hash(*h*(·); Concat(*SK_GS_,ID_U_,X,T_U_,α,ID_S_n__,Y,T_S_,T_G_*)))
(*ω*, Hash(*h*(·);Concat(*ID_U_*,Hash(*h*(·);*ID_S_n__,h*(*x* ⊕ *y*)),*T_U_*)))	(SN12) *K_SU_* ← Scalar-multiplication(*r_S_, X*)
(SN4) Generate-nonce(*r_S_*)	(SN13) *τ* ← Hash(*h*(·); Concat(*Y, T′_S_, δ, K_SU_*))
(SN5) *Y* ← Scalar-multiplication(*r_S_, P*)	(SN14) *sk* ← Hash(*h*(·); Contat(*X, Y, K_SU_*))
(SN6) *β* ← Hash	(SN15) Send(*U*, {*Y, T_S_, T′_S_, δ, τ*})
(*h*(·); Concat (*SK_GS_, ID_U_, X, T_U_, α, ω, ID_S_n__, Y, T_S_*))	(SN16) Update(*Y, T_S_,T′_S_,δ, τ*)

**Table 8.** Local sets specification for principal *GW*.

**Principal *GW***	
POSS(GW) = {*x, y, h*(*x* ⊕ *y*), *SK_GS_*}	Phase 2
BEL(*GW*) = {#(x), #(*y*), #(*h*(*x* ⊕ *y*)), #(*SK_GS_*)}	(GW9) Receive(*S_n_*, {*ID_U_, X, T_U_, α, ω, ID_S_n__, Y, T_S_, β*})
BL(*GW*)	(GW10) Check-freshness(*T_S_*)
Phase 1	(GW11) Check (*β, Hash*(*h*(·); Concat(*SK_GS_, ID_U_, X, T_U_, α, ω, ID_S_n__, Y, T_S_*)))
(GW1) Received(*U*, {*ID_U_*, ${\bar{pw}}_{U}$)	
(GW2) *K_U_* ← Scalar-multiplication(Hash(*h*(·); Concat(*ID_U_, x*)), *P*)	(GW12) *X′* ← Scalar-multiplication(Hash(*h*(·); Concat(*ID_U_, x*)), *X*)
(GW3) *A_U_* ← XOR ( ${\bar{pw}}_{U}$, *h*(*x* ⊕ *y*))	(GW13)
(GW4) *B_U_* ← Hash(h(·); Concat(*ID_U_*, ${\bar{pw}}_{U}$, *h*(*x* ⊕ *y*)))	Check(*α*, Hash(*h*(·); Concat(*ID_U_, ID_S_n__, X, X′, T_U_, ω*)))
(GW5)	(GW14) *γ* ←
*W_U_* ← XOR(Hash(*h*(·); Concat(*ID_U_*, ${\bar{pw}}_{U}$)), *K_U_*)	Hash(*h*(·); Concat(*SK_GS_, ID_U_,X,T_U_,α,ID_S_n__,Y,T_s_,T_G_*))
(GW6) Send(*U*, {*A_U_, B_U_, W_U_, h*(·)})	(GW15) *δ* ← Hash(*h*(·);Contat(*ID_U_, X, X′, T_U_, ID_S_n__, Y, T_S_*))
(GW7) Update(*A_U_, B_U_, W_U_, h*(·))	(GW16) Send(*S_n_*, {*T_G_, γ, δ*})
(GW8) Forget(*ID_U_*, ${\bar{pw}}_{U}$, *A_U_, B_U_, K_U_, W_U_*)	(GW17) Update(*T_G_, γ, δ*)
